# Racial Equity and U.S. Law

**DOI:** 10.1089/heq.2022.29022.aje

**Published:** 2023-01-20

**Authors:** Alan Jenkins

**Affiliations:** Harvard Law School, Cambridge, Massachusetts, USA.

**Keywords:** constitution, civil rights, law, equal protection

## Abstract

History and experience teach us that our Constitution and laws can be instruments of racial discrimination and oppression as well as tools for advancing freedom and equality. The substance of our laws matters, and there is much to be learned from innovative policies and legal strategies around the country. In addition to laws promoting health equity and racial justice at the state, tribal, and local levels, a new federal Executive Order has the potential to drive major positive change if fully and properly implemented. Just as important, however, is linking legal advocacy with dynamic social movements, shrewd communication strategies, and courageous civic leadership that insists on transformative change. In this article, I briefly recount the turbulent relationship between law and equity in our nation; discuss the elements that can lead to major progress through law; and recommend specific steps that different actors can take to move an equity and opportunity agenda forward.

## The Turbulent Relationship Between Racial Equity and American Law

Although a thorough recounting of the history of racial justice and injustice through U.S. law is beyond the scope of this brief, it is important to highlight some foundational aspects of this paradoxical relationship. Our founding documents articulated the ideals of freedom and equality, even as they institutionalized slavery, facilitated policies of genocide toward Native Americans, and excluded women and many others from full citizenship. The chief arbiter of American law, the U.S. Supreme Court, repeatedly strengthened these systems of oppression, notoriously asserting in *Dred Scott v. Sandford* that Black people, whether enslaved or free, were “so far inferior that they had no rights which the white man was bound to respect,”^1^ and declaring indigenous people “savages” unworthy of retaining lands that they had occupied since time immemorial.^2^

A bloody Civil War and hard-fought amendments to our Constitution formally incorporated fundamental principles of equal justice into American law and extended the franchise to include Black men. In the post-Civil War period, known as the first Reconstruction, Congress passed civil rights, voting, and anti-terror laws and deployed federal troops to enforce them in the insurrectionist southern states.^3,4^ As many as 2000 Black Americans were elected to public office at the local, state, and federal levels.^5^ New integrated statehouses adopted universal public education and other economic and social reforms^6^ as freedom and equality began to take hold.

But after just 12 years of uneven enforcement, the nation turned its back on equal justice and the law again became predominantly an instrument of racial subordination and White supremacy.^3,7^ Although formalized “Jim Crow” segregation and violence prevailed in the South, Northern states instituted their own forms of discrimination and racial separation in housing, employment, education, and other walks of life.^8^

As the nation grew more diverse through immigration, its laws were used to further reinforce racial hierarchy. Discriminatory legislation governing immigration and citizenship excluded and denigrated Latinx and Asian American communities. The well-documented and extensive history includes the deportation to Mexico of one million *United States citizens* of Mexican descent,^9–11^ and the internment of Japanese Americans—both citizen and noncitizen—in concentration camps during World War II; an atrocity upheld by the U.S. Supreme Court in the notorious *Korematsu* case.^12^

At the same time, our government continued a devastating pattern of broken treaties, forced relocation, dispossession, and mass violence toward Native Americans, even while ostensibly recognizing that Indian Tribes constitute sovereign entities with the right to self-governance.^13^ The courts were active participants in this injustice, justifying the stripping of Tribal Nations' land and autonomy based on a White supremacist “Doctrine of Discovery.”^2^

Overt, institutionalized discrimination continued long into the 20th Century. As just one poignant example, distinguished scholar Richard Rothstein has painstakingly documented how explicit government policies at the local, state, and federal levels entrenched residential segregation in America that continues today.^14^ Perhaps most notably, the Federal Housing Administration (FHA) explicitly mandated that housing proposals that included Black residents would not be approved for crucial federal subsidies. Rothstein notes that the FHA went so far as to require the denial of loans for all-White housing projects located *near* neighborhoods where Black families lived because doing so would “risk infiltration by inharmonious racial groups.”^15^

The period from the end of World War II through the early 1970s saw significant positive changes in our laws, policies, and practices—a period that is often called America's “Second Reconstruction.”^16–18^ Driven by the activism of everyday people of color, the aftermath of the Holocaust and Great Depression, Cold War competition for the hearts and mind of Global South nations, and other factors,^19^ our Constitution, courts, and laws became more frequent instruments of equity and positive change.

Supreme Court decisions during this era struck down many aspects of formal overt discrimination in cases such as *Brown v. Board of Education*^20^ (segregated schools), *Shelley v. Kraemer*^21^ (racially restrictive housing covenants), *Harper v. Virginia Board of Elections*^22^ (poll taxes), and *Loving v. Virginia*^23^ (anti-miscegenation). Although the changes in American Indian law were less monumental, important cases such as *United States v. Mazurie*^24^ and *Williams v. Lee*^25^ gave more substance to the promise of tribal sovereignty; acknowledging that Indian nations and their citizens have the power “to make their own laws and be ruled by them.”

During the same era, the U.S. Congress passed historic legislation formally outlawing discrimination and segregation in employment,^26^ voting,^27^ housing,^28^ education,^29^ public accommodations,^30^ and federally funded programs.^31^ The Indian Self-Determination and Education Assistance Act^32^ provided for more explicit, formal, and systematized tribal roles in programs affecting Native Americans. The Immigration and Nationality Acts of 1952^33^ and 1965,^34^ respectively, removed race (though not ancestry) as an exclusion for immigration and naturalization and replaced a national origin quota system with one emphasizing family reunification and skilled immigrants.

The Executive Branch, too, became a frequent (if sometimes reluctant) ally of racial equity during this period. Republican and Democratic presidents deployed federal troops and monitors to enforce civil rights.^35,36^ President Lyndon Johnson's administration, in particular, played an active role in proposing and enforcing civil rights legislation, creating innovative federal programs and regulations, appointing pro-civil rights judges, and utilizing the bully pulpit to advance justice and equality.^37^

Another important development in the post-war period was the creation, with U.S. leadership, of the international system of human rights. The Universal Declaration of Human Rights, which Eleanor Roosevelt and key civil rights leaders helped to advance,^38^ articulates not only civil and political rights such as free speech and suffrage, but also economic, social, and cultural rights such as the right to education and the right to economic security.^39^ In the years that followed, the world's nations developed a robust and particularized body of international human rights laws and institutions.^40^

The legal transformations during this period were far from perfect, much less complete. Even at their zenith, they left untouched many fundamental systems of racial inequity, such as socioeconomic discrimination,^41^ biased policing,^42^ the death penalty,^43^ and the ability of Congress to terminate or diminish tribes' sovereign status.^44^ The United States, moreover, never fully embraced the racial equity dimensions of its international human rights obligations.^45^ As a general proposition, however, the Second Reconstruction era represented a major step in the direction of racial equity through law.

That era came to a close by the 1980s, as backlash against racial equity gains led to a wave of elected leaders, judicial nominees, and institutions committed to undoing civil rights gains and policies.^46^ The U.S. Supreme Court, for its part, began issuing decisions making it extremely difficult to prove or remedy discrimination. Among many other rulings, the Court required proof of subjective discriminatory intent to invalidate racially inequitable policies,^47^ disallowed many governmental efforts to address societal discrimination,^48^ refused to remedy intentional school segregation that traverses city and suburban districts,^49^ allowed capital punishment despite the proven influence of racial bias,^50^ immunized police officers from liability for large categories of unconstitutional conduct,^51^ and invalidated a key provision of the Voting Rights Act as beyond Congress's authority.^52^ Most legal observers believe that the current Supreme Court, the most conservative in a century,^53^ is poised to further hamper progress toward racial equity.

The unfinished business of racial equity through law has profound implications today. The legacy of governmentally fostered residential segregation persists, for example, cutting communities of color off from networks of opportunity such as quality schools, jobs, and health care.^54^ After decades of improvement in school integration and educational equity, America's schools are now largely separate and unequal based on race and poverty.^55^ The Supreme Court's decision decimating the Voting Rights Act unleashed a tidal wave of laws restricting electoral participation by people of color.^56^ The lack of either strong civil rights laws or judicial accountability has enabled the perpetuation of a criminal justice system in which racial bias heavily influences policing, prosecution, and sentencing.^57^ And the United States' rejection of economic and social human rights has left unchecked rising inequality and economic insecurity.

The legacy of broken promises and disrespect for tribal sovereignty has devastating consequences too extensive to fully recount here. As just one contemporary example, Indian law expert Mary Kathryn Nagle (Cherokee Nation of Oklahoma) has explained how denigration of tribal sovereignty has allowed a high concentration of environmental hazards in Indian country. Regarding the Dakota Access Pipeline, which was vigorously opposed by the Standing Rock Sioux Tribe, Nagle writes that “[o]il now flows through a pipeline that not only creates a threat to the drinking water of millions downstream, but has also destroyed burials and desecrated sacred sites.”^58^

## Hope on the Horizon

Despite—or, perhaps, because of—this bleak assessment of racial inequity and its effects, an important positive development offers hope for the future: the rise of social movements. Equitable changes in law have come about only when demanded through activism, organizing, and engagement. The Civil Rights Movement, the Women's Rights Movement, the LGBTQ+ Movement, and others have successfully demanded laws that upheld their own rights while advancing equity and opportunity for all. The law followed in their wake, not the other way around.^59^

Today, we live in an era of movements, the majority of which seek transformative racial, economic, and social justice. The demand for racial justice and police accountability that erupted after the unjust killings of George Floyd, Breonna Taylor, and Ahmaud Arbery engaged between 15 and 26 million Americans in demonstrations alone, making it the largest social movement in U.S. history.^60^ The early decades of the 21st century have also seen mass movements for economic justice (Occupy Wall St.), women's rights (the Women's March and #MeToo), immigrant rights (the Dreamers), LGBTQ+ equality (marriage equality and transgender rights), and indigenous rights and environmental justice (Dakota Access Pipeline), among others. These intersectional movements have not only engaged tens of millions of people, but also have helped to develop new narratives and activist infrastructure that can be redeployed toward new challenges. They offer hope for meaningful change, even in dark times.

## Pathways Forward

Despite a challenging legal and policy environment, there are important opportunities to expand racial equity in the short, medium, and long term. I discuss examples below while noting that they are merely a cross section of extant possibilities. What they have in common is the potential to improve the lives of large communities of people while embedding lasting principles of equity in our laws and practices.

### Short term

A number of policies adopted as a result of racial justice protest in 2020 and 2021 present new opportunities for systemic change. At the federal level, one of the most immediate is Executive Order 13985, “Advancing Racial Equity and Support for Underserved Communities Through the Federal Government,” signed by President Biden on his first day of office.^61^ Building on existing civil rights statutes and regulations, the Order declares that “[b]ecause advancing equity requires a systematic approach to embedding fairness in decision-making processes, executive departments and agencies … must recognize and work to redress inequities in their policies and programs that serve as barriers to equal opportunity.”^62^

Among other measures, the order requires the head of every federal agency to “assess whether underserved communities and their members face systemic barriers in accessing benefits and opportunities available pursuant to [selected] policies and programs”; to “consult with members of communities that have been historically underrepresented in the Federal Government and underserved by, or subject to discrimination in, Federal policies and programs” and to “produce a plan for addressing…any barriers to full and equal participation in [those] programs….”^63^

Because it is an Executive Order rather than a statute or judicial ruling, this policy can be repealed or ignored by subsequent administrations. For at least the next several years, however, it represents a potential sea change in the way that federal funds are invested and in the proactive implementation of key civil rights laws. It also affords communities of color a platform for effectively demanding equity and opposing discrimination. That change is especially important right now, given the recent passage of the $1 trillion Infrastructure Investment and Jobs Act^64^ and the $1.9 trillion American Rescue Plan Act of 2021.^65^ Both are subject, from their inception, to the equity Executive Order.

At the time of this writing, some 90 federal agencies have produced Equity Action Plans to implement the Executive Order in important aspects of their activities.^66^ As just three examples:

- The Department of Transportation is implementing a “proactive agency review of the potential discriminatory impact of grantees' proposed activities *before* awarding federal funds…and empowering community voices in transportation decision-making.”^67^ This change offers communities of color a platform to both challenge harmful transportation projects that may result in displacement or blight and encourage clean transportation options that connect residents to employment, education, health care, and other opportunities.
- The Department of Justice is increasing access by “culturally specific, community based organizations” to federal grants, training, and technical assistance. This includes programs aimed at interrupting community violence and preventing violence against women.^68^ This change offers an important opportunity for communities of color to shape and demand approaches that enhance community safety, prevent harm, and uphold the values of equal justice and accountability.
- The Environmental Protection Agency is integrating the principle of “Community Science”—which it defines as “research and science conducted by the community on its own behalf to inform decision-making”^69^—into its own research and program implementation processes. This change will enable communities of color around the nation to study, document, and demand action to address the environmental harms that disproportionately affect their neighborhoods due to bias and discrimination.^70^

The Biden administration has also taken several steps regarding tribal sovereignty and indigenous peoples' rights that offer opportunities for greater equity. Under the leadership of Secretary Deb Haaland, the first Native American to serve as a cabinet secretary, the Department of the Interior has, among other actions, issued a plan to integrate tribal input into the Interior Department's decision-making processes.^71^ The new approach has already led to tangible advances in sovereignty; the Interior Department's Bureau of Land Management and the U.S. Forest Service recently signed a historic cooperative agreement with five tribes—the Hopi Tribe, the Navajo Nation, the Ute Mountain Ute Tribe, the Ute Indian Tribe of the Uintah and Ouray Reservation, and the Pueblo of Zuni—giving those tribes a substantive role in managing the Bears Ears National Monument.^72^

In an important step toward accountability and healing, the Interior Department has also launched the Federal Boarding School Initiative to address the legacy and intergenerational trauma of Indian boarding schools in the United States. On May 11, 2022, the Initiative released Volume 1 of its investigative report.^73^ As the letter from Assistant Secretary Bryan Newland transmitting the report explains, “[t]his report places the Federal Indian boarding school system in its historical context, explaining that the United States established this system as part of a broader objective to dispossess Indian Tribes, Alaska Native Villages, and the Native Hawaiian Community of their territories to support the expansion of the United States.”^74^

The same day on which the report was released, Secretary Haaland announced plans to meet with survivors of Indian boarding schools around the country in a tour called “The Road to Healing.” According to the Department of the Interior, the year-long tour will “allow American Indian, Alaska Native, and Native Hawaiian survivors of the federal Indian boarding school system the opportunity to share their stories, help connect communities with trauma-informed support, and facilitate collection of a permanent oral history.”^75^

These Executive Branch actions, although important, will not lead to significant change on their own. They require community organizing, strategic communications, research, advocacy, ethical leadership, and mass community participation to be effective. Communities must be informed, engaged, and resourced, for example, to insist on federally funded transportation projects that connect communities and avoid inequitable environmental hazards and displacement. Activists must be prepared for the potential backlash and “NIMBYism” that often meets equitable and integrated development projects.^76^

Philanthropies must be willing to support grassroots civic participation as well as research and advocacy. That said, these new policies offer the chance to do things differently (and better) in the context of trillion-dollar investments. And utilizing them can ingrain equity principles in law while building community leadership. To facilitate broad public engagement, the Executive Order also establishes an “Interagency Working Group on Equitable Data” to gather, coordinate, and ensure disaggregation of federal data sets by race, ethnicity, gender, disability, income, veteran status, or other relevant demographic characteristics.^77^

### Medium term

Although Congress remains deadlocked on pressing equity legislation,^78^ a range of policy and practice changes are available to state, tribal, and municipal lawmakers to advance equity in the medium term. At their best, these efforts improve the lives of everyday people while advancing structural changes and precedents.

In the voting sphere, for example, a number of states are tackling racially inequitable obstacles to voting, even as other states are erecting discriminatory barriers.^79^ New York State, for example, recently passed the John R. Lewis Voting Rights Advancement Act,^80^ which, among other things, creates new protections against voter intimidation, deception, and obstruction within the state; expands language assistance for voters with limited English proficiency; and requires local governments with records of discrimination to prove that certain voting changes will not hinder voters of color *before* those changes can go into effect.

Several months earlier, New York City adopted legislation granting an estimated 808,000 noncitizen immigrants the right to vote in citywide and local elections for positions such as mayor, public advocate, city council, and borough president.^81^ These laws are important because they both provide a voice to people who would otherwise be shut out of decisions that affect them and establish important equitable principles in policy and practice.

Another significant development is the pursuit of reparative laws by state and local governments to address historical wrongs that have continuing harm. Most noteworthy is the decision by Evanston, Illinois to provide reparations for harm caused by “discriminatory housing policies and practices and inaction on the city's part.”^82^ The city's program directs revenue from taxes on cannabis to support home ownership, home improvement, and mortgage programs for Black individuals who lived in Evanston between 1919 and 1969 and direct descendant individuals harmed by discriminatory housing policies or practices during that time period.^83^ The Evanston program appears to be the first of its kind in the nation and offers a potential model for other jurisdictions.

At Harvard University (where I serve on the Law School faculty), the Presidential Committee on Harvard and the Legacy of Slavery recently released an extensive report documenting the university's ties to slavery and anti-Black discrimination and calling for a range of reparative actions.^84^ The University accepted the Task Force's recommendations in full and established a $100 million fund to implement them.^85^

Although each has its limitations, these programs offer measures of acknowledgment, accountability, and repair to communities while instituting the principle of reparation in American law and policy. They warrant engagement by communities of color, and some are worthy of adaptation elsewhere.

On the environmental justice front, a number of Tribal Nations are developing responses to climate change that embody each tribe's values, reaffirm tribal sovereignty, and are responsive to the particular vulnerability of indigenous communities to climate change that flows from forced displacement and discrimination.^86^ As a 2020 article reported, for example, “[t]he Swinomish Indian Tribal Community completed a climate change health impact assessment and action plan by tailoring an existing framework to Swinomish-specific health values, definitions, and priorities,” while the Pala Band of Mission Indians “is developing climate and health communication and outreach materials tailored to community needs, including culture-based psychosocial resilience strategies.”^87^ These efforts can be adapted to individual tribes' values and circumstances, alongside broader advocacy to stem the drivers of climate change.

### Long term: A third reconstruction

Ultimately, the breadth, depth, and longevity of racial bias and institutional discrimination in our country require transformative legal changes that will take many years to achieve and must transcend the limitations of today's judicial and political environment. The needed transformation amounts to what many scholars and advocates have termed a “Third Reconstruction.”^88–91^ Similar to the fundamental changes to our Constitution wrought by the First Reconstruction (1865–1877), and the Civil Rights revolution of the 1950s and 1960s, finishing the job of equity and opportunity for all will require vision, struggle, and sacrifice. I focus here on needed changes in constitutional interpretation and jurisprudence, while recognizing that these are elements of a much larger change agenda.

First and crucially, a reconstructed jurisprudence must recognize that the core purpose of the 13th, 14th, and 15th Amendments is to end racial subordination and supremacy. The courts must review policies that implicate race with an understanding of history, context, and circumstances—striking down those that perpetuate the subjugation or supremacy of any group and upholding those that move us closer to equal citizenship. That includes heeding Justice Harry Blackmun's poignant statement in *Regents of the University of California v. Bakke*^92^ that “[i]n order to get beyond racism, we must first take account of race. There is no other way. And in order to treat some persons equally, we must treat them differently. We cannot—we dare not—let the Equal Protection Clause perpetrate racial supremacy.”^93^

Second, our courts and laws must acknowledge that the Civil War and Reconstruction Amendments worked a fundamental change in the relationship between the federal government, the states, and the people—not merely a modest adjustment of the Constitution to extinguish the institution and trappings of slavery. They must reject what Professor Peggy Cooper Davis and colleagues describe as a “Confederate Narrative” and embrace a “People's Narrative” focused on human rights and dignity.^94^

That means recognizing that Congress has broad authority to protect human rights, ensure due process, and guarantee the privileges and immunities of citizenship, and that so-called “states' rights” arguments cannot hamper that authority. It means not only reconsidering the Court's modern-day decisions eviscerating key provisions of the Voting Rights Act^52^ and the Violence Against Women Act,^95^ but also acknowledging broad federal power to address discrimination at the state, local, and private levels, as well as nationally. As the First Justice Harlan said in a powerful dissenting opinion, “[i]f the constitutional amendments be enforced according to the intent with which, as I conceive, they were adopted, there cannot be, in this republic, any class of human beings in practical subjection to another class with power in the latter to dole out to the former just such privileges as they may choose to grant.”^96^

Third, the courts and Constitution must recognize that conscious intentional bigotry is only one of the sources of unlawful, remediable discrimination in the modern era. Research and experience make clear that subconscious bias and institutional discrimination also perpetrate inequity at a mass scale and of a kind that the Civil War Amendments were designed to prevent.^97^ As a practical matter, this means overturning the ill-considered discriminatory intent standard that the Court adopted in *Washington v. Davis*^47^ and, instead, adopting more sophisticated disparate impact and institutional bias standards that reflect human nature and modern brain science.^98^

A fourth change that is needed to align our laws with the goal of systemic equity is robust incorporation of the international human rights laws that the United States helped to craft in the aftermath of the Holocaust, World War II, and the Great Depression. The Convention on the Elimination of all forms of Racial Discrimination (CERD), to which the United States is a party, includes significantly more effective approaches to racial inequity than do most U.S. laws. CERD defines “discrimination,” for example, as “any distinction, exclusion, restriction or preference based on race, colour, descent, or national or ethnic origin which has the purpose or effect of nullifying or impairing the recognition, enjoyment or exercise, on an equal footing, of human rights and fundamental freedoms in the political, economic, social, cultural or any other field of public life.”^99^

The Inter-American Commission on Human Rights recognizes the important notion of “intersectionality”; the idea that “discrimination on the basis of race is inextricably linked with other factors including sex, ethnicity, national origin, religion or belief, gender identity and expression, sexual orientation, health, age, disability, and class, among others.”^100^ As noted earlier, the full body of international human rights laws provide even more fulsome guarantees of equity and human dignity.

Whether through a change in the current Court's jurisprudence, a change in its membership, or clarifying amendments to our Constitution, this long-term transformation is needed to achieve true racial equity under the law.

## Conclusion

Through most of U.S. history, our Constitution, laws, and courts have been instruments of racial injustice rather than of equity or fairness. Yet, through sacrifice and activism, two historic Reconstructions have offered a vision of a just nation supported by equitable laws and policies. While working toward a transformative Third Reconstruction, communities of color and their allies have important opportunities today to improve individuals' lives while advancing principles of positive systemic change.

## Abbreviations Used

CERD: Convention on the Elimination of all forms of Racial Discrimination
FHA: Federal Housing Administration
